# Evo-Devo of the Human Vertebral Column: On Homeotic Transformations, Pathologies and Prenatal Selection

**DOI:** 10.1007/s11692-012-9196-1

**Published:** 2012-08-18

**Authors:** Clara M. A. ten Broek, Alexander J. Bakker, Irma Varela-Lasheras, Marianna Bugiani, Stefan Van Dongen, Frietson Galis

**Affiliations:** 1 Group of Evolutionary Ecology, University of Antwerp, Groenenborgerlaan 171, 2020 Antwerp, Belgium; 2 The John Innes Centre, Norwich Research Park, Colney, Norwich, NR4 7UH UK; 3 Gulbenkian Institute of Science, Oeiras, Portugal; 4 Department of Pathology, VU Medical Centre, 1081 BT Amsterdam, The Netherlands; 5 Naturalis Biodiversity Center, Darwinweg 2, 2333 CR Leiden, The Netherlands; 6 Department of Cell and Developmental Biology, University of East Anglia, Norwich, NR4 7TJ UK

**Keywords:** Anterior-posterior patterning, Developmental constraints, Congenital abnormalities, Mammals, Presacral vertebra number

## Abstract

Homeotic transformations of vertebrae are particularly common in humans and tend to come associated with malformations in a wide variety of organ systems. In a dataset of 1,389 deceased human foetuses and infants a majority had cervical ribs and approximately half of these individuals also had missing twelfth ribs or lumbar ribs. In ~10 % of all cases there was an additional shift of the lumbo-sacral boundary and, hence, homeotic transformations resulted in shifts of at least three vertebral boundaries. We found a strong coupling between the abnormality of the vertebral patterns and the amount and strength of associated malformations, i.e., the longer the disturbance of the vertebral patterning has lasted, the more associated malformations have developed and the more organ systems are affected. The germ layer of origin of the malformations was not significantly associated with the frequency of vertebral patterns. In contrast, we find significant associations with the different developmental mechanisms that are involved in the causation of the malformations, that is, segmentation, neural crest development, left-right patterning, etc. Our results, thus, suggest that locally perceived developmental signals are more important for the developmental outcome than the origin of the cells. The low robustness of vertebral A-P patterning apparent from the large number of homeotic transformations is probably caused by the strong interactivity of developmental processes and the low redundancy of involved morphogens during early organogenesis. Additionally, the early irreversibility of the specification of the A-P identity of vertebrae probably adds to the vulnerability of the process by limiting the possibility for recovery from developmental disturbances. The low developmental robustness of vertebral A-P patterning contrasts with a high robustness of the A-P patterning of the vertebral regions. Not only the order is invariable, also the variation in the number of vertebrae per region is small. This robustness is in agreement with the evolutionary stability of vertebral regions in tetrapods. Finally, we propose a new hypothesis regarding the constancy of the presacral number of vertebrae in mammals.

## Introduction

The spectacular diversification of the vertebrate body plan since the Ordovician is to an important extent due to the evolvability of the segmented vertebral column. The shape, size and number of these segments can be changed relatively easily, which provides for a body plan with a large degree of freedom (Vermeij 1973; see also Lauder and Liem 1988; Galis 2000). The long necks of plesiosaurs, the fused sacral vertebrae of the sacrum in terrestrial amniotes and the long thorax of snakes are but a few of the many striking examples of innovation in the vertebral column. These innovations involve modifications of the number, size and shape of vertebrae of different regions. Any change in the number of one or another type of vertebrae almost by definition involves homeotic transformations, emphasizing the key role of homeotic transformations in evolution (Bateson 1894). It is often assumed that changes of the number of vertebrae in a region do not necessarily require homeotic transformations and can be solely the result of meristic changes, however this is not true, except for vertebrae in the tail region that is formed last. Homeotic transformations are unavoidably involved, because of the preceding sequential anterior-posterior (A-P) generation of the somites, coupled to a simultaneous A-P patterning over these somites under the control of A-P signalling gradients (Diez del Corral et al. 2003; Aulehla and Pourquie 2010; Mallo et al. 2010). This implies that if there is a change in the number of precaudal vertebrae, for instance an increase from 7 to 9 cervical vertebrae, this is caused by a homeotic transformation of the somites that contribute to the 8th and 9th vertebrae (i.e. an anteriorization of the identity from thoracic to cervical), regardless of whether the total number of vertebrae changes or not. Homeotic transformations do not necessarily imply a change in the total number of vertebrae, however they frequently go hand in hand (Varela-Lasheras et al. 2011), which is not surprising given the developmental link between axial lengthening and axial patterning, which are both under the control of the same A-P gradients of morphogens (Dubrulle et al. 2001; Dubrulle and Pourquie 2004).

**Fig. 1:**
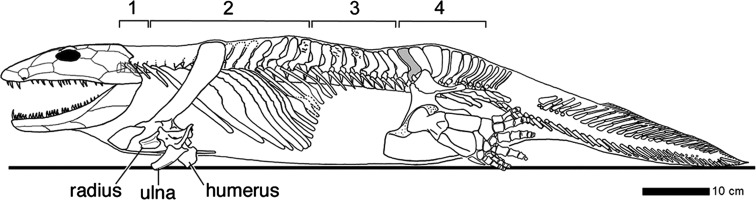
The vertebral column of the stem-tetrapod Ichthyostega shows a remarkably mammal-like pattern with cervical (*1*), thoracic (*2*), lumbar (*3*), sacral (*4*) and caudal vertebrae (reprinted with permission from Ahlberg et al. 2005)

Alongside the remarkable evolutionary diversification, there has also been impressive conservation of the vertebral column. One of the strongest examples is the conservation of the different vertebral regions of tetrapods. The early tetrapod *Ichthyostega* already had a surprisingly mammal-like vertebral column with recognizable cervical, thoracic, lumbar, sacral and caudal regions (Ahlberg et al. 2005; Fig. 1) and when looking more widely among early tetrapods we find that cervical, sacral and caudal regions always can be distinguished (Ahlberg, pers. comm.). In most mammals these regions and their anterior-posterior order have been faithfully maintained, but in extant mammals only the thoracic vertebrae still possess ribs. In Mesozoic mammals the ribs on the lumbar vertebrae were lost twice independently and once the presence of ribs on all lumbar vertebrae was regained (in *Akidolestes*; Luo et al. 2007). These subsequent losses and the regain of ribs for *all* vertebrae of the lumbar region emphasize the importance of regional specification of the vertebral column, alongside the specification of individual vertebrae within regions. The global roles that *Hox* genes are found to have in mediating the A-P vertebral patterning also points at the importance of regional specification (Wellik and Capecchi 2003; Carapuco et al. 2005; Mallo et al. 2010; Vinagre et al. 2010).

The number of vertebrae within specific vertebral regions is also highly constrained in many vertebrate taxa. For instance, mammals as a rule have seven cervical vertebrae and turtles 8 cervical vertebrae and 10 thoracic (also called dorsal) vertebrae (e.g. Starck 1979; Zug et al. 2001). The constancy of the number of cervical vertebrae in mammals probably results from stabilizing selection against changes of this number (Galis 1999; Galis and Metz 2003). In a study on early human mortality we found that the selection is indirect and caused by a strong coupling of such changes with deleterious pleiotropic effects (Galis et al. 2006). Changes of the number of cervical vertebrae were found to be exceptionally common in humans (~7.5 % of all conceptions), but strongly selected against: virtually all individuals die before the age of reproduction (Galis et al. 2006). The high incidence of cervical ribs in deceased human foetuses was recently confirmed by a study of Furtado et al. (2011). In addition, several paediatric cancers are significantly associated with cervical ribs, further increasing the strength of selection (Schumacher et al. 1992; Galis and Metz 2003). Thus, human data support that pleiotropic constraints (Hansen and Houle 2004) are at the root of the evolutionary conservation of the number of cervical vertebrae in mammals.

Moreover, we have proposed that the unavoidability of such pleiotropic effects is due to the strong interactivity during the early developmental stage when the number of cervical vertebrae is determined (Galis et al. 2006). This determination happens as part of the early anterior-posterior patterning of the paraxial mesoderm, mediated by the well-known *Hox* genes (e.g. Kessel and Gruss 1991; Kmita and Duboule 2003; Wellik and Capecchi 2003; Carapuco et al. 2005; Deschamps and van Nes 2005; Woltering and Durston 2008; Vinagre et al. 2010). The strong interactivity of this early organogenesis stage presumably results from the interactions between the patterning of the three body axes and interactions of these axial patterning processes and simultaneously occurring morphogenetic processes, such as the division and migration of cells, somitogenesis and the active maintenance of the bilateral symmetry of somites (e.g. Diez del Corral et al. 2003; Cordes et al. 2004; Kawakami et al. 2005; Vermot and Pourquie 2005; Galis et al. 2006; Aulehla and Pourquie 2010; Durston et al. 2011). This strong interactivity precludes an effective modularity. As a result, even slight disturbances of the early organogenesis stage cause deleterious pleiotropic effects (Galis and Metz 2001); see also Sander (1983) and Raff (1996). Hence, the low effective modularity not only appears to cause the conservation of the number of cervical vertebrae, but that of the entire stage. During this stage a large number of traits of the conserved body plan are determined, including the number of limbs, digits, lungs, kidneys, eyes and ears. Therefore, we proposed that pleiotropic constraints and stabilizing selection play a major role in the evolutionary conservation of body plans (Galis and Metz 2007).

We recently tested our hypotheses on the importance of pleiotropic constraints and stabilizing selection in a study on sloths and manatees, the only mammals that as a rule have an exceptional number of cervical vertebrae (Varela-Lasheras et al. 2011). We compared sloths and manatees with sister species that usually have a normal number of cervical vertebrae, anteaters, armadillos (sister taxa of sloths), dugongs and hyraxes (sister taxa of manatees). We found a surprisingly high number of defects in the skeletons of sloths and manatees and also in those aberrant individuals of sister taxa that had an abnormal number of cervical vertebrae. The high number of skeletal abnormalities that we found in sloths and manatees supports our pleiotropy hypothesis. The abnormalities are also in agreement with our hypothesis that low metabolic and activity rates reduce the usual stabilizing selection, allowing the breaking of the pleiotropic constraints on changes of the number of cervical vertebrae.

To better understand the developmental mechanisms involved in the association of homeotic transformations of cervical or other vertebrae and malformations in other parts of the body we studied 1,389 deceased human foetuses and infants in the VU University Medical Centre in Amsterdam. We analysed radiographs to identify homeotic transformations along the entire vertebral column and we identified the associated malformations in different organ systems from obduction reports of pathologists. We investigated the relationship between the severity of the disturbance of the vertebral A-P pattern and the severity of the associated malformations. The detailed record of malformations allows us to study the influence of the disturbance of different morphogenetic processes on vertebral patterning and the influence of disturbances of particular germ-layers. These data shed further light on the high interactivity of early organogenesis, which is crucial for the vulnerability and low developmental robustness of this highly conserved stage (Galis et al. 2002).

## Materials and Methods

### Subjects

Since 1980, all foetuses and infants that were presented for medical examination at the VU Medical Centre have standardly been radiographed both ventrally and laterally (23 mA, 70–90 kV, 4–12 s, Agfa [Mortsel, Belgium] Gevaert D7DW Structurix films).

For this study we analysed all radiographs made between 1990 and 2009, including 1,389 foetuses and infants. In the analysis, we only included foetuses where the ossification centres could be reliably detected, taking the visibility of the ossification centres of the phalanges as a proxy. In practice we included only foetuses older than 13 weeks in our study, of which some of the foetuses had developed a cervical rib, which is comparable to the earliest age of detection of cervical ribs at 14 weeks of Noback and Robertson (1951) and McNally et al. (1990). At least two different observers independently analysed the radiographs for variation in the vertebral column, without prior knowledge of the autopsy reports (however, several congenital abnormalities can be observed in the radiographs). In total, 98 foetuses were excluded from analysis because of insufficient ossification. Radiographs that were too difficult to interpret or where the interpretation differed between the observers were excluded as well (229 foetuses). Difficulties in interpretation of vertebral variations were due to either insufficient contrast or because the clavicula, scapula, maxilla, or teeth were obstructing the view of possible cervical ribs. We examined 589 males and 460 females (aged: 13–92 weeks, mean: 26.6 ± 10.4 weeks), 13 foetuses had an unknown sex because gonads and/or genitals were undeveloped or undifferentiated. Foetuses from abortions induced for medical reasons (lethal abnormalities) have been included in this research.

Data on ethnicity were not available, but the patient population of the VU Medical Centre is predominantly Caucasian. Gestational ages were provided by the gynaecologist, based on the time since the first day of the last normal menstruation period.

### Vertebral Variations

#### Variations in Identity and Number of Vertebrae

Ribs on the seventh vertebra were considered to be a transitional cervico-thoracic vertebra except for the rare cases where a rib was present that was longer than half the length of the adjacent thoracic rib. Enlarged transverse processes (apophysomegaly) of the seventh cervical vertebra were considered to be rudimentary cervical ribs fused to the transverse process when the length of the transverse process exceeded that of the first thoracic vertebra (Pionnier and Depraz 1956; Brewin et al. 2009; Bots et al. 2011; see also McNally et al. 1990; Redenbach and Nelems 1998; Merks et al. 2005). Ribs on the most anterior and most caudal thoracic vertebra were considered to be rudimentary when their length was less than half that of the rib of the adjacent thoracic vertebra. Regarding the number of vertebrae of a vertebral region, we counted transitional vertebrae as having half the identity of the two neighbouring regions, e.g. transitional cervico-thoracic vertebrae were counted as half cervical/half thoracic. The lumbo-sacral transition was not as easy to interpret as the transition at other boundaries, as in most individuals the sacral vertebrae were not yet fused to each other or to the ilium. It is, therefore possible that we have underestimated transitional lumbo-sacral vertebrae and aberrations of the number of presacral vertebrae. Nonetheless, the shape and position of the vertebrae also provides information that allows evaluation of their identity.

#### Severity Scale of Variations of the Vertebral Formula

**Fig. 2:**
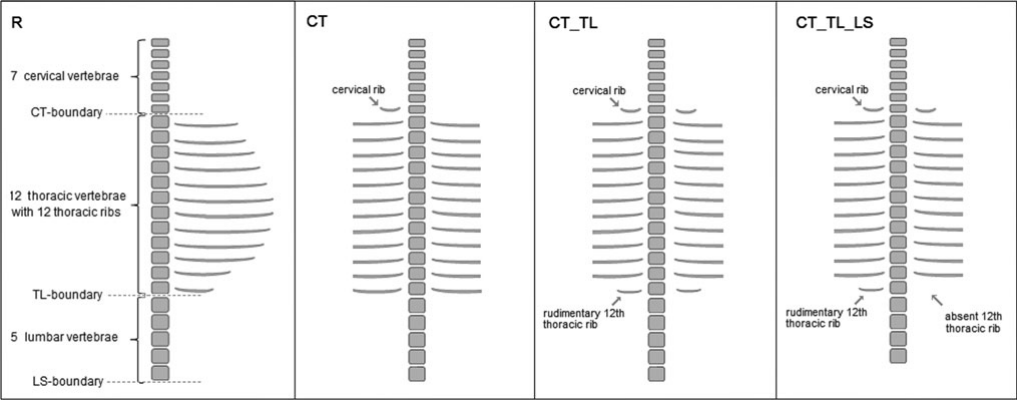
Schematic representation of common variations of vertebral patterns and indication of values on the severity scale. From *left* to *right*: Regular pattern with 7 cervical vertebrae, 12 thoracic vertebrae with ribs and 5 lumbar vertebrae (pattern R, severity scale value 0); Pattern with only a shift of the cervico-thoracic boundary: a cervical rib on the seventh vertebra (cervical ribs are also often bilateral) and the number of thoracic and lumbar vertebrae is regular (Pattern CT, severity scale value 6); Pattern with a shift of the cervico-thoracic and thoracolumbar boundary: cervical ribs on the seventh vertebra, rudimentary twelfth thoracic ribs and a normal number of lumbar vertebrae (pattern CT_TL, severity scale value 8); Pattern with shifts of the cervico-thoracic, thoraco-lumbar and lumbo-sacral boundary: cervical ribs, rudimentary twelfth rib (unilateral) and four instead of five lumbar vertebrae (pattern CT_TL_LS, severity scale value 9). This pattern has only 23 presacral vertebrae

We scored changes of the vertebral formula on a severity scale that reflects our expectation of the seriousness of the disturbance of the vertebral anterio-posterior (A-P) patterning, based on both the A-P position of the changes and the extent of the changes along the A-P axis (see Galis et al. 2006). More anterior changes (cervical ribs and rudimentary first ribs) are considered to be more deleterious, based on the strength of the selection, which is considerably stronger than the selection against more posterior changes (Galis et al. 2006). Furthermore, we assume that changes involving two or more boundaries reflect a longer developmental disturbance than those involving one boundary and those involving three boundaries reflect a longer developmental disturbance than those that involve two boundaries, based on teratological results that show that the timing of the treatment is related to the A-P position of homeotic transformations of vertebrae (e.g. Menegola et al. 1998; Kawanishi et al. 2003; Rengasamy and Padmanabhan 2004). For these reasons, we have scored a regular (R) vertebral column with 7 cervical, 12 thoracic and 5 lumbar vertebrae as 0; a change of the number of lumbar vertebrae and, hence of the number of 24 presacral vertebrae, without other changes (LS) as 1, lumbar ribs and absent or rudimentary twelfth ribs (TL) as 3, lumbar ribs and absent or rudimentary twelfth ribs with a changed number of presacral vertebrae (TL_LS) as 4, a cervical rib or rudimentary or absent first rib (CT) as 6, a cervical rib or rudimentary or absent first rib with a changed number of presacral vertebrae (CT_LS) as 7, a cervical rib or rudimentary or absent first rib with in addition an absent or rudimentary twelfth rib, or lumbar rib (CT_TL) as 8 and a cervical rib or rudimentary or absent first rib with an absent or rudimentary twelfth rib, or lumbar rib and with a changed number of presacral vertebrae (CT_TL_LS) as 9 (Fig. 2).

### Diagnosis of Malformations and Abnormalities

Standard autopsy reports were made by pathologists and filed in a national pathological archive (PALGA; www.palga.nl). We examined the reports and categorised the different congenital abnormalities into distinct groups of malformations in the different organ systems: bronchopulmonary malformations (BP), cardiovascular malformations (CV), craniofacial malformations (CF), digestive system malformations (DS), limb defects (LD), muscular system defects (MS), nervous system defects (NS), skeletal malformations (SK), urogenital malformations (UG), and ventral body wall defects (VBW). Not all individuals were in good conditions due to maceration, resulting in the score “non-available” for some of their organ systems. When autopsy was not approved, internal organ systems were also scored as “non-available”.

**Fig. 3:**
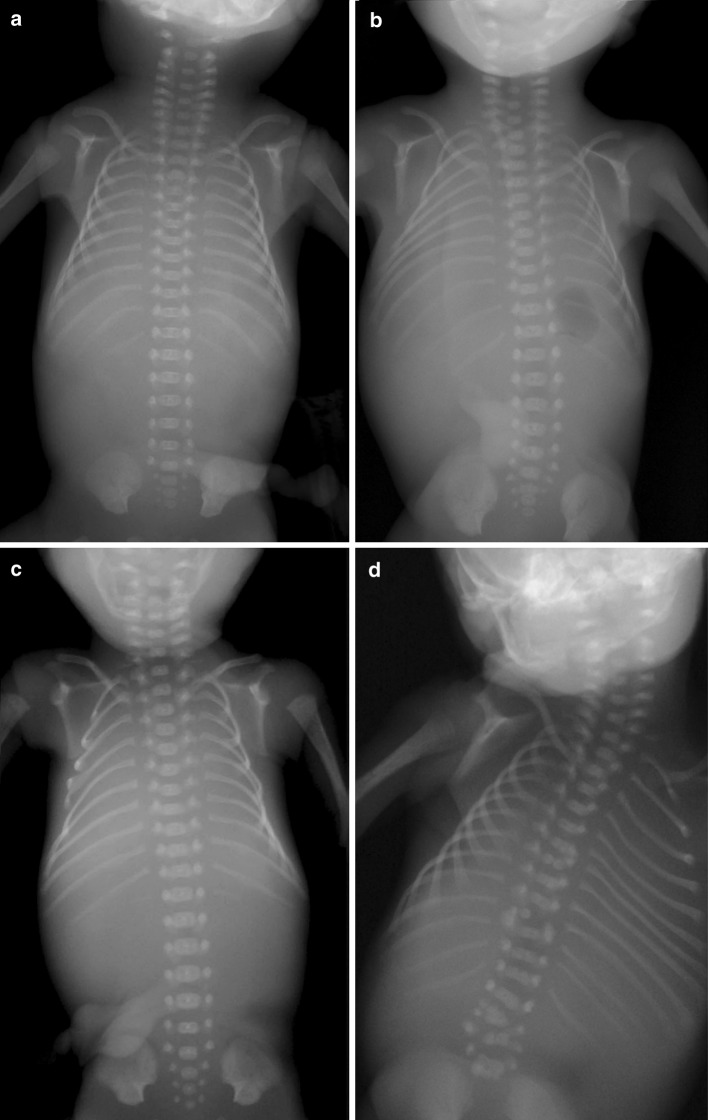
Radiographs of deceased fetuses with different vertebral patterns. **a** Regular pattern with 7 cervical, 12 thoracic and 5 lumbar vertebrae (pattern R). **b** Fetus with bilaterally rudimentary first thoracic ribs (Pattern CT, with cervico-thoracic boundary change). **c** Fetus with unilaterally a cervical rib and bilaterally absent twelfth thoracic ribs (Pattern CT_TL, with changes of the cervico-thoracic and thoraco-lumbar boundary). **d** Fetus with bilaterally cervical ribs and full-sized lumbar ribs (Pattern CT_TL, with changes of the cervico-thoracic and thoraco-lumbar boundary). The vertebrae and ribs are highly irregular with hemivertebrae and fused ribs, these represent segmentation defects

As far as possible we scored malformations only in one category, by distinguishing between primary and secondary causes, with only primary causes counted in the analyses: Tracheo-esophageal (T-E) fistula’s were counted as primary for the digestive system and secondary for the bronchopulmonary system, because both the trachea and the esophagus develop from the primary gut and, in addition, atresia of the esophagus often accompanies the T-E fistula; skeletal dysplasias were scored as primary for the skeletal system and secondary for limb and craniofacial malformations; Holoprosencephaly as primary for the nervous system and secondary for craniofacial malformations; limb reduction and polydactyly as primary for the limbs and secondary for skeletal malformations; hypoplasia of the lung secondary when caused by skeletal dysplasia or diaphragmatic hernia and oligohydramnion; all malformations caused by hypoxic stress were scored as secondary as well (Fig. 3).

### Statistical Analysis

To explore the relationships between variations of the vertebral column on the severity scale on the one hand and malformations in different organ systems on the other, we first performed a simple correspondence analysis (R, ca package, Nenadic and Greenacre 2007). Analysis was conducted on two different types of contingency to explore effects of changes on the LS-boundary: in the first table we grouped the differences in presacral number, resulting in four severity categories of increasing severity(R & LS, TL & TL_LS, CT & CT_LS, and CT_TL & CT_TL_LS grouped together); in the second table we did distinguish between differences in presacral vertebrae number on the severity scale, and thus retained our eight severity levels.

Next we used generalized linear modeling (glim) techniques with logit link function and binomial error structure to test the statistical significance of patterns in the correspondence analysis, because an analysis based on the marginal totals of the contingency table alone can easily lead to incorrect conclusions about associations (see also Galis et al. 2006). Variation of the vertebral column on the severity scale was tested against the occurrence of (a) the different types of malformations, (b) affected germ layers and (c) developmental processes. We used the severity scale with eight levels in the models, because the correspondence analysis showed that changes on the LS-boundary retained valuable information and should remain in the model. Sex and age were also included in the model and treated as factors but were not significant as expected (Galis et al. 2006). The frequency distribution of the different types of vertebral variation did not vary with gestational age (χ^2^ = 0.16, df = 1, *p* = 0.87) nor sex (χ^2^ = 0.16, df = 1, *p* = 0.62). We checked for overdispersion of the data relative to the model and a significance level of 95 % was used throughout.

All analyses were conducted in R (R Developmental Core Team 2011).

## Results

### Correspondence Analyses

**Fig. 4:**
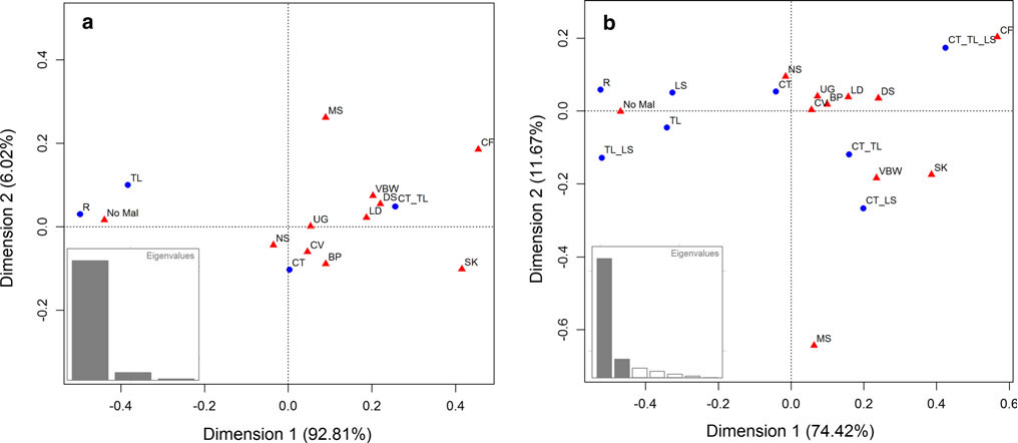
Correspondence analyses showing the associations between malformations of the different organ systems and vertebral patterns. Eigenvalues are indicated. **a** The vertebral patterns included changes at the cervico-thoracic, thoraco-lumbar and lumbo-sacral boundaries. **b** Changes at the more difficult to evaluate lumbo-sacral boundaries were left of out this analysis, which leads to a very high explained variance of the first axis (92.8 vs. 74.43 %). In both analyses the group with no malformations (No-Mal) clusters with the most normal vertebral patterns (R, LS, TL, TL-LS) and the groups with craniofacial and skeletal malformations (CF and SK) cluster with the most abnormal patterns (CT_TL_LS and CT_TL)

We investigated the associations between the malformations of the different organ systems and the vertebral variations at the C-T and T-L boundary (disregarding changes at the L-S boundary) with a correspondence analysis. The results show that the first dimension (x-axis) accounts for 92.8 % of the variation (Fig. 4a). This dimension reflects from left to right an increasing severity of variation in vertebral columns. No transformations (R) and the least disturbed patterns (changes at the T-L boundary) cluster with the no-malformations group. On the right side, the most changed vertebral patterns (changes of both the C-T and T-L boundaries) cluster most closely with malformations of the craniofacies, skeleton, digestive system, limbs and ventral body wall. The other malformations are located in between and thus appear to associate less strong with the severity scale of vertebral abnormalities. Dimension 2 (plotted on the y axis) explains only a small fraction of the variation (6.0 %), with the small group of muscular anomalies (n = 27) at the most extreme position.

In the second correspondence analysis we investigated the associations between the malformations of the organ systems and the vertebral variations at three vertebral boundaries, including changes at the L-S boundary. Most variation was again explained by dimension 1 (74.4 %) and in general a similar pattern emerged along this axis as in the first analysis, with from left to right a change from the normal and least disturbed vertebral patterns to the most abnormal pattern which had changes at all three boundaries, C-T, T-L and L-S (Fig. 4b). The left-to-right order of malformations was also approximately the same as in the first correspondence analysis, with the no-malformations group clustering with the regular and least disturbed vertebral patterns. On the right, the most abnormal pattern, in this case with changes at three rather than two boundaries, only clustered strongly with the craniofacial and skeletal malformations, whereas the ventral body wall defects and digestive system malformations were found in between this pattern and those with changes at two of the three boundaries. Dimension 2 explained 11.8 % of the variation and again the muscular malformations were a contrasting group. This group was furthest away on this y-axis from the craniofacial anomalies and the group with changes at all three vertebral boundaries.

### Agreement Between the Results of the Correspondence Analysis and Our Severity Scale

The changes at the LS-boundary which were included in the second analysis in contrast to the first analysis were not clustering that closely together, indicating that changes at this third boundary hold valuable information and should be considered in further analysis. In the correspondence analysis the variation of the vertebral column are considered as separate groups rather than values on an severity scale, but from left to right basically the same pattern emerged as it were an severity scale with values. The most abnormal vertebral pattern is found furthest away from the normal vertebral pattern and also the position of the other patterns was a good approximation of our severity values. Only value 7 and 8, that both represented changes at two boundaries (C-T and L-S boundaries for 7, versus C-T and T-L boundaries for 8), showed a reverse order. This supports our choice of values on the severity scale as a reflection of the severity of the disturbances.

### Homeotic Transformations of the Vertebral A-P Pattern

**Fig. 5:**
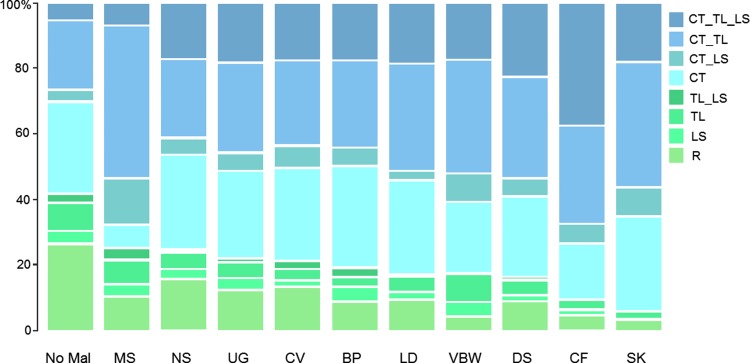
Frequencies of vertebral patterns for fetuses with malformations of different organ systems. Vertebral patterns are indicated with R, LS, TL, TL_LS, CT, CT_LS, CT_TL, CT_TL_LS, see Fig. 2 for an explanation. Malformations are indicated for the different organ systems: *MS* Muscular System, *NS* Nervous System, *UG* Urogenital System, *CV* Cardiovascular System, *BP* Bronchopulmonary System, *LD* Limb Defects, *VBW* Ventral Body Wall Defects, *DS* Digestive System, *SK* Skeletal Malformations, *CF* Craniofacial Malformations, *No Mal* No Malformations. The group with no malformations has the highest incidence of regular patterns and the groups with skeletal and craniofacial malformations has the lowest incidence of regular patterns

**Fig. 6:**
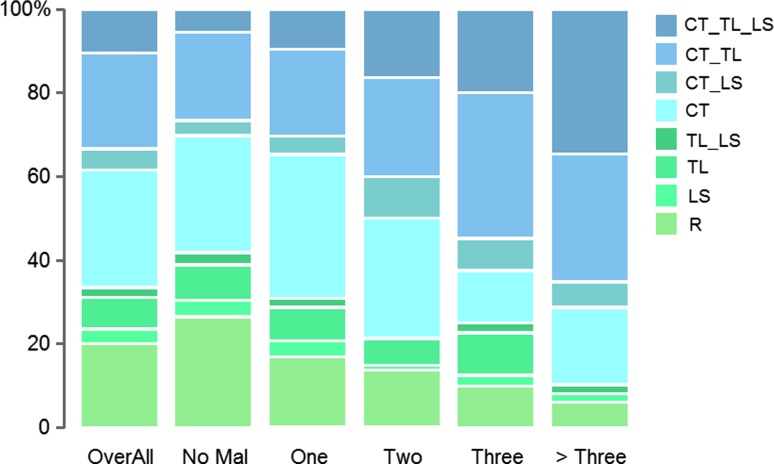
Frequencies of vertebral patterns for fetuses with a different number of affected organ systems. Vertebral patterns are indicated with R, LS, TL, TL_LS, CT, CT_LS, CT_TL, CT_TL_LS, see Fig. 2 for an explanation. The number of organ systems affected: No Mal—no organ system affected, one to three or more than three organ systems affected, overall-represents the entire population of our dataset. The group with no malformations has the largest frequency of regular patterns and the group with more than three organ systems affected has the largest frequency of the most abnormal vertebral pattern (CT_TL_LS)

**Fig. 7:**
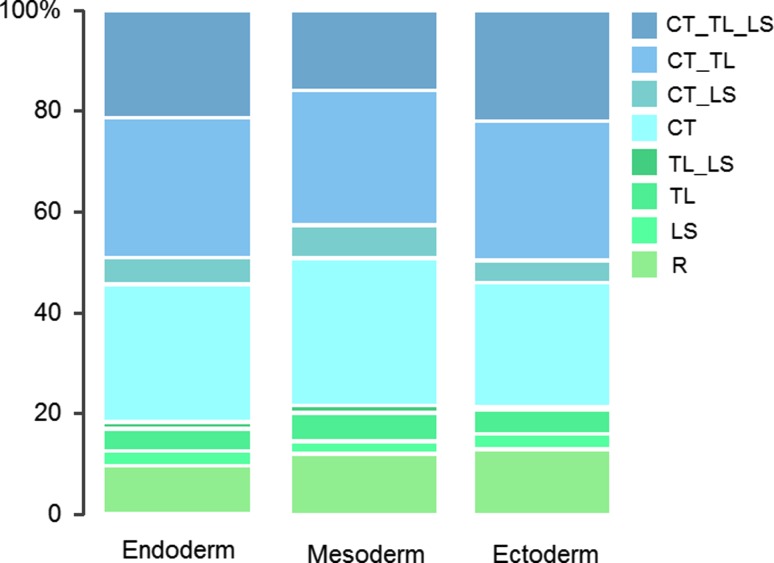
Frequencies of different vertebral patterns for fetuses with malformations of a particular germ-layer, i.e. endoderm, mesoderm, ectoderm. Vertebral patterns are indicated with R, LS, TL, TL_LS, CT, CT_LS, CT_TL, CT_TL_LS, see Fig. 2 for an explanation. There is no significant effect of the germ-layer of origin on the frequencies of vertebral patterns

**Fig. 8:**
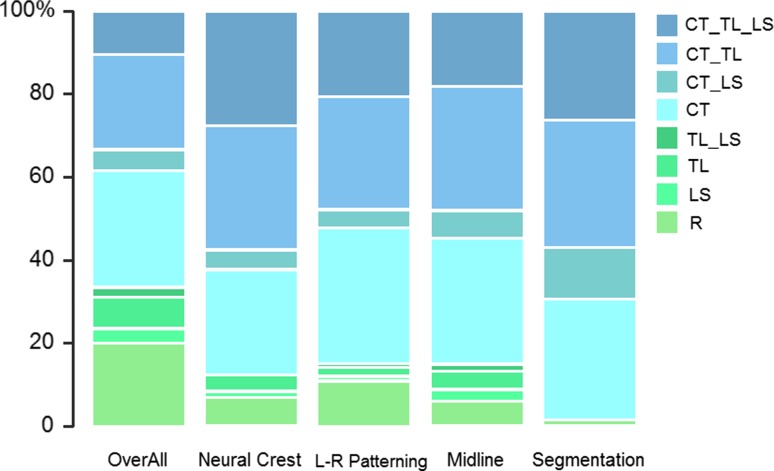
Frequencies of different vertebral patterns for fetuses with malformations that involve disturbances of particular morphogenetic processes, i.e. neural crest-involved malformations, left-right and midline patterning defects, segmentations defects. Overall-represents the entire population of our dataset. Vertebral patterns are indicated with R, LS, TL, TL_LS, CT, CT_LS, CT_TL, CT_TL_LS, see Fig. 2 for an explanation. The vertebral patterns differ significantly for malformations that involve different morphogenetic processes (see text)

We found that 20.6 % of the 1,062 fetuses and infants of which the vertebral pattern could be analysed had a regular pattern (R in Figs. 2, 3a, light green shade in Figs. 5, 6, 7, 8). In 3.4 % of the cases we found a shift at the L-S boundary and in 8.6 % a shift at the T-L boundary with or without a change at the L-S boundary (resp. LS and TL & TL_LS, green shades). The majority of the fetuses and infants (67.4 %) showed evidence of homeotic transformations at the C-T boundary (blue shades, CT & CT_LS, CT_TL & CT_TL_LS), Almost all these cases had unilateral or bilateral cervical ribs on the seventh vertebra, a posteriorization of the identity of the seventh vertebra, which results in an anterior shift of the C-T boundary. More rarely rudimentary or absent first ribs (9.8 %) were involved, which resulted in a posterior shift of the C-T boundary. Half of the cases with a shift of the C-T boundary (33.4 %) also had homeotic transformations at the thoraco-lumbar boundary (CT_TL & CT_TL_LS), usually rudimentary or absent twelfth ribs, resulting in an anterior shift of the T-L boundary. In 10.6 % we found homeotic transformations at all three investigated boundaries: the C-T, T-L and L-S boundary (CT_TL_LS), which may be the result of one large homeotic shift, but can also be explained by several homeotic shifts that affected at least these three boundaries.

### Malformations of Different Organ Systems, Affected Germ Layers and Developmental Processes

Cardiovascular malformations were the most common type of malformations with 23.6 % of evaluated cases (n = 216), followed by malformations of the urogenital system (18.1 %, n = 168), the nervous system (15.1 %, n = 121), digestive system (13.7 %, n = 110), skeleton (12.7 %, n = 115) and the limbs (13.7 %, n = 146). Craniofacial malformations were considerably more rare (6.0 %, n = 64) and malformations of the muscles and ventral body wall were the least common malformations (3.0 %, n = 28 and 2.2 %, n = 23).

The germ layer of origin was malformed in 41.3 % of all the cases for the mesoderm (n = 439), in 15.5 % for the endoderm (n = 165), and in 15.3 % of the cases for the ectoderm (n = 163). Midline patterning defects were present in 17.0 % of all the cases (n = 181), abnormal neural crest development in 12.2 % (n = 130), left-right patterning defects in 8.7 % (n = 92), and segmentation defects in 6.1 % of the cases (n = 65).

### Associations Between Malformations and Vertebral Patterns

#### Positive Association Between Congenital (Minor and Major) Malformations and Severity of Abnormality of Vertebral Patterns

Significant positive associations between the seriousness of variations of the vertebral column on the severity scale and the prevalence of malformations were observed for all organ systems, except for the muscular malformations: bronchopulmonary system (n = 68, slope: 0.12, SE: 0.05, *p* < 0.01); cardiovascular system (n = 216, slope: 0.12, SE: 0.03, *p* < 0.001); ventral body wall (n = 23, slope: 0.18, SE: 0.09, *p* < 0.05), craniofacies (n = 64, slope: 0.36, SE: 0.07, *p* < 0.001), digestive system (n = 110, slope: 0.19, SE: 0.04, *p* < 0.001), limbs (n = 146, slope: 0.17, SE: 0.03, *p* < 0.001), nervous system (n = 121, slope: 0.07, SE: 0.03, *p* < 0.05), skeleton (n = 115, slope:0.31, SE: 0.05 *p* < 0.001), urogenital system (n = 168, slope: 0.11, SE: 0.03, *p* < 0.001 and the muscular system (n = 28, slope: 0.11, SE: 0.07, *p* = 0.12).

The slope was steepest for craniofacial and skeletal malformations and the lowest for nervous system malformations followed by muscular and urogenital malformations. This pattern is also confirmed by the distribution of vertebral abnormalities in fetuses with different types of malformations (Fig. 5). The occurrence of a regular vertebral pattern is the least common for skeletal and craniofacial malformations, whereas the most abnormal pattern is the most common for the craniofacial malformations.

In addition, there is a significant positive association between the seriousness of the vertebral variations on the severity scale and the number of different affected organ systems. The slope increases with more affected organ systems: No malformed organ system (slope = −0.02, SE = 0.09, *p* = 0.80), one malformed organ system (slope = 0.06, SE = 0.09, *p* = 0.56), two malformed organ systems (slope = 0.14, SE = 0.10, *p* = 0.17), three malformed organ systems (slope = 0.18, SE = 0.11, *p* = 0.09), and four or more malformed organ systems (slope = 0.34, SE = 0.13, *p* < 0.05). Pairwise comparisons showed that all slopes are (slightly) significantly different from one each other (0.07 < *p* < 0.001), except for two and three malformed organ systems, (*p* = 0.55, Fig. 6). Figure 6 further shows that the frequency of a regular vertebral pattern (light green shades) decreases with the number of organ systems that is affected (significantly negative correlation, R = −0.97, *p* = 0.006). In contrast, the incidence of the most abnormal patterns increased significantly (dark shades of blue, R = 0.96, *p* = 0.009).

In contrast, the association between the abnormality of vertebral patterns and the frequency of individuals with no malformations was significantly negative (n = 357, slope: −0.11, *p* < 0.001). Figure 6 showed that the occurrence of a regular vertebral pattern was the most common in the no-malformations.

### No Effect of Germ Layer of Origin

The frequency of occurrence of the different vertebral patterns is remarkably similar for malformations in different germ layers (Fig. 7). We found increasing slopes for the three germ layers: endoderm (n = 165, slope = 0.16, SE = 0.04, *p* < 0.01), mesoderm (n = 439, slope = 0.16, SE = 0.03, *p* < 0.001) and ectoderm (n = 163, slope = 0.12, SE = 0.04, *p* < 0.01), but the interaction between the severity scale and the different germ layers was not significantly different from each other (χ^2^ = 1.101, df = 2, *p* = 0.58).

### Significant Effect of Morphogenetic Processes

Significant positive associations between the severity of the vertebral pattern changes and the frequency of disturbances of specific morphogenetic processes were found: left-right patterning defects (n = 92, slope = 0.16, SE = 0.04, *p* < 0.01), midline patterning defects (n = 181, slope = 0.20, SE = 0.04, *p* < 0.001), neural crest (n = 130, slope = 0.22, SE = 0.05, *p* < 0.001) and segmentation defects (n = 65, slope = 0.39, SE = 0.08, *p* < 0.001). Two by two comparisons showed that segmentation defects had a significantly higher slope on the severity scale than left-right pattering and midline defects (t = 2.447, *p* < 0.05 and t = 2.188, *p* < 0.05 respectively). We observed a slight, (but) only marginally significant, difference between the slopes of neural crest-involved and segmentation defects (t = 1.810, *p* = 0.08). There is no significant difference between left-right patterning and midline defects, which have a very similar distribution of vertebral patterns (t = 0.586, *p* = 0.56) and also no significant difference between neural crest-involved and left-right patterning defects (t = 0.923, *p* = 0.37) and between neural crest-involved and midline defects (t = −0.44, *p* = 0.66) was found. Figure 8 shows that segmentation defects hardly ever co-occur with a normal vertebral pattern. In an additional analysis we excluded segmentation defects in a separate analysis from the organ systems to compare the effects between skeletal malformations with and without segmentation defects. We again found a positive association for skeletal malformations when we excluded the segmentation defects (slope = 0.21, SE = 0.05, *p* < 0.001, n = 49).

Neural crest-involved malformations and segmentation defects co-occur most often with the two most abnormal vertebral patterns, but neural crest-involved malformations have a lower incidence of cervical ribs and of cervical ribs with an associated shift of the lumbo-sacral boundary. Left-right and midline patterning defects have the highest co-occurrence of isolated cervical ribs.

## Discussion

### High Frequency of Homeotic Transformations and Shifts

Cervical ribs are known to be extremely frequent in humans that die before or around birth, which shows that there is extremely strong selection against these vertebral variations that result from a homeotic transformation of the seventh vertebra from a cervical into a thoracic identity (Galis et al. 2006; Bots et al. 2011; Furtado et al. 2011). Our present results for a large dataset of more than 1,000 deceased fetuses and infants of which the vertebral pattern could be analysed in detail confirm these earlier findings. In roughly two-thirds of the cases (67.42 %) we found cervical ribs. This indicates that in a large majority of humans that die before the first year, the early A-P patterning of the paraxial mesoderm has been disturbed. The high incidence of disturbances highlights the vulnerability of the early organogenesis stage (Sander 1983; Raff 1996; Galis and Metz 2001; Sander and Schmidt-Ott 2004).In about half of the cases with cervical ribs (~33 % of all individuals) there has been a prolonged disturbance of development, because homeotic transformations were also observed at the later specified T-L boundary. In ~10 % we found homeotic changes at three boundaries, the C-T, T-L and L-S boundary, suggesting a homeotic shift over most of the vertebral column and a long disturbance of development. Homeotic shifts that are restricted to the T-L and L-S boundaries were more rare and comparable with frequencies recorded in the general population (Galis et al. 2006). Selection against shifts of T-L and L-S boundaries is, thus, considerably weaker than selection against shifts of the C-T boundary (Galis et al. 2006), in agreement with the weaker constraint against changes of the number of thoracic and lumbar vertebrae.

In only one in five of the deceased fetuses and infants did we find a vertebral column with a regular pattern, i.e. seven cervical, twelve thoracic and five lumbar vertebrae. Homeotic transformations are, thus, extremely common and arguably the most common malformations in humans, considering that ~15 % of clinically recognized pregnancies end in still births. The high prevalence of homeotic transformations of vertebrae implies a low effective robustness of A-P vertebral patterning, in particular in the cervical and thoracic regions.

### Severity of Vertebral Aberrations Coincides with Severity of Malformations

The severity of the disturbances of the vertebral patterns was strongly associated with the severity of other malformations, measured in several ways. Firstly, in the correspondence analysis we found clustering of the groups with a normal vertebral pattern and the no-malformations and clustering between the groups with specific malformations and various abnormal vertebral patterns (Fig. 4a, b). Secondly we found positive association between the abnormality of the vertebral pattern (reflected in the values on our severity scale, Fig. 6) and the number of organ systems affected. Furthermore, we found a positive association between the abnormality of the vertebral pattern and the prevalence of malformations for each organ system (except for the muscular malformations, where the positive association was not significant, Fig. 5). Finally, we found a significantly negative association between the abnormality of the vertebral patterns and the prevalence of no malformations in individuals.

### Skeletal and Craniofacial Malformations Associated with the Largest Homeotic Shifts

Skeletal and craniofacial malformations appear to be particularly strongly associated with the most abnormal vertebral patterns (Fig. 5). Cleft lip and cleft palate are the most commonly found craniofacial malformations; other common craniofacial malformations include ear tags, abnormal position of the ears and nose and reduced lower jaws (not caused by a deficiency of amniotic fluid). Other neural crest-involved malformations also are highly associated with abnormal vertebral patterns, especially those also found in the head like holoprosencephaly (classified as nervous system malformations). Segmentation defects and skeletal dysplasias are the most common skeletal defects. Especially segmentation defects are strongly associated with abnormal vertebral patterns (see also Fig. 8), but there is also a positive association with skeletal malformations when we excluded segmentation defects.

Earlier we also found skeletal abnormalities such as ossification defects and fusions of vertebrae to be extremely common in sloths and manatees (Varela-Lasheras et al. 2011). Sloths and manatees have vertebral patterns that are similar to the most abnormal ones in our dataset, i.e. homeotic shifts at all vertebral boundaries. We hypothesized that sloths and manatees can survive with these serious abnormalities due to their extremely slow lifestyle. We did not find craniofacial malformations in sloths and manatees, probably these malformations are more strongly selected against than the skeletal (non-craniofacial) malformations, as the former will more strongly affect feeding capacity.

### Perception of Developmental Signals more Important than Germ Layer of Origin

There was no significant effect of germ layer of origin on the vertebral patterns associated with malformations (Fig. 7). In contrast, there was a significant effect for the different developmental processes involved in the causation of malformations, segmentation (somitogenesis), neural crest development, left-right and midline patterning. This suggests that the locally perceived developmental signals are more important for the developmental outcome than the origin of the cells. For instance, the influence of organizers may be more important than origin of germ layer of cells. During the patterning of the A-P axis in the paraxial mesoderm, the opposing gradients of Fgf/Wnt and retinoic acid (RA) and the segmentation clock are important organizers for a rather large part of the embryo (e.g. Diez del Corral et al. 2003; Moreno and Kintner 2004; Iimura et al. 2009; Aulehla and Pourquie 2010). Later on progressively more and more signalling centres appear, organizing smaller and smaller developmental fields (in the limb, heart, brain, etc.) leading to a more compartmentalized development. Within these smaller developmental fields intense signalling between tissues continues to occur, which may further explain the absence of an effect of the germ layer of origin of the malformations.

### Close Association Between A-P Vertebral Patterning and Other Morphogenetic Processes

Segmentation defects virtually always co-occur with homeotic transformations. These results highlight the earlier found coupling between somitogenesis and A-P patterning of the paraxial mesoderm (Zakany et al. 2001) (Dubrulle et al. 2001; Cordes et al. 2004; Iimura et al. 2009; Durston et al. 2011). Furthermore, we found that homeotic transformations of vertebrae are virtually always characterized by asymmetric transitional vertebrae, as is a general phenomenon for homeotic transformations in natural mutants in amniotes and in transgenic mice (Varela-Lasheras et al. 2011). For instance, the size of rudimentary ribs was almost always unequal, be they cervical, thoracic or lumbar. Rudimentary ribs were also frequently found on only one side. This confirms the strong coupling between A-P patterning of the paraxial mesoderm and the preservation of left-right symmetry (Kawakami et al. 2005; Vermot et al. 2005; Vermot and Pourquie 2005; Sirbu and Duester 2006; Echeverri and Oates 2007; Vilhais-Neto et al. 2010; Varela-Lasheras et al. 2011).

The highly abnormal vertebral patterns that are associated with craniofacial malformations are in agreement with the high degree of abnormality of vertebral patterns found for neural crest-involved malformations. Our data suggest that in particular neural crest-involved malformations of the head are associated with the most abnormal vertebral patterns (craniofacial malformations and holoprosencephaly), more so than neural crest-involved malformations in the trunk. Possibly, this is because the craniofacial skeleton and cartilage is mainly built from neural crest-derived cells, whereas in the trunk neural crest-derived cells only constitute a minor part of the organs to which they contribute (e.g. the heart). Malformations that are caused by faulty left-right patterning are found in a wide variety of organs, e.g. the heart, lungs, intestines, spleen, brain) and because other malformations are also common for these organs, the influence on vertebral patterns is less clear. Left-right and midline patterning defects appear to result in very similar frequencies of the different vertebral variations. This is not so surprising because earlier findings show a coupling between midline and left-right patterning defects (Danos and Yost 1996; Meno et al. 1998; Przemeck et al. 2003; Lee et al. 2010; Lenhart et al. 2011) which are often found to co-occur.

These results are, thus, in agreement with developmental studies that indicate a high interactivity between A-P patterning of the paraxial mesoderm and other patterning and morphogenetic processes that occur during early organogenesis. The positive association of abnormal vertebral patterns with malformations of all different organ systems further support the high interactivity.

### Low Effective Robustness of Vertebral A-P Patterning

An analysis of teratological data of rodents shows that early organogenesis is more vulnerable to disturbances than other stages due to intense and global interactiveness (Galis and Metz 2001). The strong associations between vertebral A-P patterning defects and defects due to a disturbance of other patterning and morphogenetic processes support our earlier findings on the interactiveness of early organogenesis and support our hypothesis that the interactivity is due to the close coordination between axial patterning and other morphogenetic processes (Galis et al. 2006). The low effective robustness of early vertebral A-P patterning can, thus, at least in part, be explained by the high global interactivity during this stage. A further contributing factor is probably the low redundancy of morphogens that are active during early organogenesis. For instance, the target genes of early *Hox* A-P patterning activity appear to have few paralogues and the same holds for inhibitors of the BMP pathway (e.g. Cobb and Duboule 2005; Preger-Ben Noon et al. 2009; Kang et al. 2010; Wellik 2011). This low redundancy presumably also explains the potentially devastating effects of inhibitors of HH, Wnt, BMP developmental pathways during early organogenesis, as in the chemically induced onset of cyclopic lambs (by cyclopmaine), and children with severely shortened limbs (by thalidomide) during the 1950 s (Sakata and Chen 2011). High interactiveness and low redundancy are, thus, expected to affect the robustness of the entire early organogenesis stage. However, these two factors cannot explain why homeotic transformations of cervical and thoracic vertebrae are so much more common than any other malformations that have their origin in a disturbance of early organogenesis. The explanation for the exceptionally high prevalence of these homeotic transformations may be that the identity of the vertebrae is not only specified early, but also becomes irreversible early, just after the formation of the somites (Forlani et al. 2003; Carapuco et al. 2005; Iimura and Pourquie 2006; Vinagre et al. 2010). Early irreversibility limits the possibilities for recovery from developmental disturbances. In this respect, it is of interest that the development of the cardiovascular system also appears to be extremely vulnerable, with not only a high incidence of cardiovascular defects among the fetuses of more than 13 weeks of gestation in our study, but also in earlier spontaneous abortions (e.g. Hoffman 1995). The most important patterning of the cardiovascular system occurs early, in association with an early start of its functioning: in humans the heart already starts beating within three weeks of conception, at a stage compatible to embryonic day 8.5 in mice (Hochgreb et al. 2003; Horsthuis et al. 2009; Bertrand et al. 2011). It is possible that, more in general, developmental robustness is constrained by early irreversibility, which in its turn may be associated with early functional demands (including patterning functions).

### High Effective Robustness of Vertebral Regions

The low effective robustness of vertebral A-P patterning contrasts with the high effective robustness of the patterning of the vertebral regions. Homeotic transformations are clearly not randomly distributed over the vertebral column: the presence and order of vertebral regions is in all cases unchanged, and the patterns of change can be best explained as resulting from one, or sometimes two, homeotic shifts of several adjacent vertebrae (frame-shifts).

The variation in the number of vertebrae per vertebral region also varies little: plus or minus one vertebra for the cervical (6–8 vertebrae) and lumbar region (4–6 vertebrae) and plus or minus two vertebrae for the larger thoracic region (10–13.5). This robustness of the vertebral regions is quite remarkable when one realizes that developmental disturbances often result in gross malformations in other parts of the body, e.g., holoprosencephaly and anencephaly, which upset the entire cranial region, or for instance the, almost complete absence of the urogenital system in combination with incompletely and abnormally formed limbs. Hence, in these two cases, the missing vertebrae where not due to disturbed patterning, but to an absence of rostral or caudal development. The strong developmental stability of the vertebral regions found in this study is in good agreement with the evolutionary stability of these regions in amniotes, highlighted by the mammal-like vertebral column of the early stem-tetrapod *Ichthyostega* (Ahlberg et al. 2005).

The robustness of the A-P order of the vertebral regions is not surprising, given the mechanisms of A-P patterning of the somites under the control of opposing A-P signalling gradients, resulting in the co-linear and overlapping A-P expression of *Hox* genes in the somites (e.g. Duboule 1994; Greer et al. 2000; Kmita et al. 2000; Kmita and Duboule 2003; Aulehla and Pourquie 2010; Durston et al. 2011). For the same mechanistic reasons, mutations are expected to have only a limited effect on the number of vertebrae in specific vertebral regions. A change in the order of the vertebral regions would require a particularly drastic disruption of the highly conserved temporal and spatial co-linearity of the *Hox* gene expression that characterizes the zoötype (Slack et al. 1993; Duboule 1994). The A-P patterning of the paraxial mesoderm occurs in close coordination with the A-P patterning of other tissues, hence a drastic disruption of A-P patterning cannot conceivably be viable in complex organisms, since this would lead to a host of deleterious pleiotropic effects (Galis and Metz 2001; Tschopp and Duboule 2011). For the same reasons evolutionary changes of the number of vertebrae of regions are expected to occur in a gradual way.

### The Number of Presacral Vertebrae and a Hypothesis on the Conservation of this Number in Mammals

The total number of presacral vertebrae varied from 22 to 26. Two exceptional cases were more extreme, but both are due to the absence of formation of part of the body. One case with serious caudal regression syndrome (CRS) in which the sacral and coccygeal vertebrae had not been formed (17 presacral vertebrae) and an acephalus twin which missed not only the head, but also several cervical vertebrae (19 presacral vertebrae). In CRS, the caudal part of the body is not formed due to a shortage of caudal cells, and in acephalic (and acardiac twins), the rostral part of the body is not formed due to de-oxygenated blood being received from the other twin. The variation of 22–26 is very similar to that found in adults, where the variation is 22–25, with 23 and 25 being common variants (Bardeen 1904; Schultz and Straus 1945; Bornstein and Peterson 1966; de Beer Kaufman 1974). This suggests that, at least in humans, prenatal selection against changes of the presacral number of vertebrae is probably limited.

In many mammalian taxa there is surprisingly little interspecific variation in the number of presacral vertebrae and the number is highly conserved, although some taxa, such as afrotherians, xenarthrans and primates, have considerable interspecific variation, (Narita and Kuratani 2005; Sanchez-Villagra et al. 2007; Varela-Lasheras et al. 2011). Narita and Kuretani (2005) have proposed that this number is conserved because of developmental constraints, as is also the case for the number of cervical vertebrae. We agree with Narita and Kuratani (2005) that developmental constraints are probably involved. Furthermore, we propose that the developmental constraints are caused by later occurring biomechanical problems that are associated with changes of the number of presacral vertebrae, and that these biomechanical problems lead to stabilizing selection against changes of this number. We expect biomechanical problems to be important because mutations for homeotic transformations of vertebrae usually lead to incomplete and asymmetric transitional vertebrae. In the case of lumbo-sacral transitional vertebrae this implies incomplete and asymmetric fusion to the sacrum. The abnormal fusion with the sacrum can cause serious biomechanical problems in mammals with functional hind limbs. In addition, shifts of the vertebral boundaries are usually not precisely matched with shifts of the adjacent tissues, including shifts of the plexuses, with nerves that run into the limbs, the brachial plexus and lumbo-sacral plexuses. This may lead to further biomechanical problems, such as compression of nerves and blood vessels (see Varela-Lasheras et al. 2011) for a discussion). In dogs and cats, lumbo-sacral transitional vertebrae are associated with an increased incidence of hip dysplasia and cauda-equina syndrome (Morgan et al. 1993; Damur-Djuric et al. 2006; Fluckiger et al. 2006; Shimali et al. 2010). In humans, they are associated with an increased incidence of intervertebral disc degeneration, degeneration of the iliolumbar ligament, scoliosis and also a narrowing of the birth canal in women (e.g. Aihara et al. 2005; Tague 2009). Our assumption that biomechanical problems are of primary importance leads us to furthermore propose that the constraints against changes of the number of presacral vertebrae will be strongest in mammalian taxa with fast-running species and weakest in slow, sturdy species. Preliminary data on fast-running and slow-moving carnivores and artiodactyla appear to support of this hypothesis (Galis et al. in prep.). In humans, running used to be important (Carrier et al. 1984; Carrier 2004), but selection against problems associated with transitional lumbo-sacral vertebrae, not unexpectedly, appears to be relaxed in present-day humans where incidences are reported from 4 to 36 % (Bron et al. 2007; Konin and Walz 2010; Apazidis et al. 2011) and intraspecific variation of the presacral number of vertebrae is considerable in fetuses as well as adults.

Biomechanical considerations can also explain why the number of thoracic and lumbar vertebrae separately is not conserved in mammals, unlike the number of thoracic plus lumbar vertebrae. The T-L boundary is not mechanically articulating with limb girdles or with other skeletal structures and therefore, we expect that shifts of this boundary will be associated with fewer deleterious pleiotropic effects than shifts of the L-S and C-T boundary. Indeed, transitional thoraco-lumbar vertebrae are not known to be associated with medical problems. Hence, stabilizing selection against shifts of the T-L boundary is expected to be weaker than that against shifts of the L-S boundary.

### Medical Applications

The extremely high prevalence of homeotic transformations and the association with malformations and early fetal deaths suggests that these variations may be of considerable diagnostic relevance (see Steigenga et al. 2006). Our data show that the accuracy with which the severity of the perturbation of embryonal A-P patterning is evaluated is increased when we include homeotic transformations at different boundaries. Changes at the C-T and T-L boundary can be detected on prenatal scans of fetuses, i.e. cervical ribs and rudimentary or absent first ribs (C-T boundary) and lumbar ribs and absent or rudimentary twelfth thoracic ribs (L–T boundary; Hershkovitz 2008). Changes at the L-S boundary cannot be detected because of the late ossification of sacral vertebrae. In many countries such scans are now routinely carried out during pregnancies. Analyses of vertebral patterns may, thus, be useful in evaluating medical risks. For instance, when common congenital anomalies are found that have highly variable outcomes, such as cleft lip and/or palate and cardiac anomalies, the additional vertebral information may help the evaluation of the seriousness of the condition. Furthermore, observing changes at the C-T boundary combined with shifts of the T-L boundary suggests that increased vigilance may be useful, given the particularly strong association of these vertebral patterns with multiple malformations. More in general, it would be good to explore the diagnostic possibilities of vertebral patterns on prenatal scans, especially in prospective studies.
